# Comments and General Discussion on “The Anatomical Problem Posed by Brain Complexity and Size: A Potential Solution”

**DOI:** 10.3389/fnana.2016.00060

**Published:** 2016-06-10

**Authors:** Javier DeFelipe, Rodney J. Douglas, Sean L. Hill, Ed S. Lein, Kevan A. C. Martin, Kathleen S. Rockland, Idan Segev, Gordon M. Shepherd, Gábor Tamás

**Affiliations:** ^1^Laboratorio Cajal de Circuitos Corticales, Centro de Tecnología Biomédica, Universidad Politécnica de MadridMadrid, Spain; ^2^Instituto Cajal, Consejo Superior de Investigaciones CientíficasMadrid, Spain; ^3^Centro de Investigación Biomédica en Red Sobre Enfermedades Neurodegenerativas (CIBERNED)Madrid, Spain; ^4^Institute of Neuroinformatics, Swiss Federal Institute of Technology in Zurich (ETH) and University of Zurich (UZH)Zurich, Switzerland; ^5^Blue Brain Project, Campus BiotechGeneva, Switzerland; ^6^Human Cell Types Department, Allen Institute for Brain ScienceSeattle, WA, USA; ^7^Department of Anatomy and Neurobiology, Boston University School of MedicineBoston, MA, USA; ^8^Cold Spring Harbor Laboratory, Cold Spring HarborNY, USA; ^9^Departments of Neurobiology, The Hebrew University of JerusalemJerusalem, Israel; ^10^The Interdisciplinary Center for Neural Computation, The Hebrew University of JerusalemJerusalem, Israel; ^11^Edmond and Lily Safra Center for Brain Sciences, The Hebrew University of JerusalemJerusalem, Israel; ^12^Department of Neurobiology, Yale School of MedicineNew Haven, CT, USA; ^13^MTA-SZTE Research Group for Cortical Microcircuits of the Hungarian Academy of Sciences, Department of Physiology, Anatomy and Neuroscience, University of SzegedSzeged, Hungary

**Keywords:** multi scale integration, dense and saturated reconstructions, predictive neuroanatomy, neuronal circuitry, synapses, connectome, synaptome

## Introduction

This article gathers together different opinions on the current status and future directions of the study of the brain, taking as a working document the article “The anatomical problem posed by brain complexity and size: a potential solution” http://journal.frontiersin.org/article/10.3389/fnana.2015.00104/full. These commentaries are followed by a section dedicated to a general discussion of the issues raised, in which all contributors participate. The authors who have contributed to this article are listed in alphabetical order. As the reader will see, there are different points of view and of course there are many other aspects that would need further discussion that have been raised by other scientists who did not participate directly. For example, Peter Somogyi made the following comment (personal communication):

[“Anatomy” is a discipline and not a biological entity that exists in nature. Hence the brain or its cells do not have anatomy; we study them with anatomical methods (usually using microscopes) while we carry out “anatomical analysis.” The brain, its nuclei, cells, and their parts are the biological entities which several disciplines study, preferably together, providing a unified description and explanation of them. We must be clear about this, and avoid terms like “anatomical properties,” “physiological properties,” or “biochemical properties” as if these somehow existed in isolation. The separate disciplines, which developed historically due to the limitation of individual human brain capacity and short life span leading to methodological and conceptual specialization, are based on sets of methods, but study the same indivisible biological entity. E.g., the synaptic current recorded by electrophysiological methods flows through the membrane that we see in the electron microscope or with the help of antibodies to synaptic ion channels in the light microscope. Accordingly, the “anatomical problem” exists because of inadequate scientific rigor in addition to methodological limitations that are often not understood, not because of “brain complexity”.]

This is just an example of the many possible different points of view when dealing with the subject of the anatomy of the brain. Thus, this article is not intended to be comprehensive, and the unavoidable limitations in the selection of comments, data, and their interpretation reflect, in many cases, the personal views and interests of the authors.

## A note on detail

### Rodney J. douglas, Kevan A. C. Martin

A century of neuroanatomical research has laid out the fundamental organization of many nervous systems, but has offered only limited insights into the functional principles that the neuronal circuits support. Now a variety of “high-throughput” methods offer to refine our understanding of the detailed neuronal circuitry -and thereby their signaling (on the basis of Francis Crick's dictum that knowledge of function follows automatically from a detailed knowledge of the structure). This quest for detailed structure flies under the flag of “Connectomics,” but in practice the “Connectome” is a grab-all that describes any kind of map of structural and functional connectivity at various scales: So the human connectome is mentioned in the same breath as the 302 neurons of the C elegans connectome. In these maps, connections between regions in space are necessarily quantized (the maps are not continuous) and the degree of quantization depends on the resolution of the experimental methods used to observe the connections, and also on the conceptual intent of their analyses. Neurons are a useful level of quantization, because they are relatively easily identifiable and because they are the cellular unit of connective interaction. More problematic are current efforts to achieve finer degrees of quantization at the level of synapses. Not only are the technical challenges much more severe, but the locations and relationships of individual synapses are much more difficult to describe and their very existence is more dynamic than their parent neurons. On the other hand, coarser quantization, say at the fMRI level, loses the specific structure of axonal connections.

If we require circuit-level understanding of brain processing, then there seems to be no possibility of avoiding detailed neuronal circuit reconstructions (see, for example Chklovskii et al., 2010). Whether a full circuit connectome is really necessary, or whether some sampled version will suffice, is currently a matter of divided opinion and the constraints of available methods Lichtman and Denk (2011); Bargmann and Marder (2013); da Costa and Martin (2013); Marblestone et al. (2013); Morgan and Lichtman (2013). “Big Data” connectomics ventures, such as the Human Brain Project (HBP), Brain Activity Map (BAM), and the Allen Institute for Brain Science, take the view that exhaustive structural and functional connectomics is necessary (Alivisatos et al., 2012; Koch and Reid, 2012; Waldrop, 2012). The most radical position is that of HBP, whose original claim was that the operation of the human brain will be understood by reverse-engineering it from a detailed reconstruction.

As has been clearly evident to all players, any attempt to measure a full circuit connectome for a human (or most any mammalian) brain quickly runs against practical limits of scale. The reasons are clear to see. Assume the following (orders of magnitude) estimates: 10^11^ neurons; say, 10^4^ synapses per neuron (10^16^ synapses overall); each synapse exist for at least 1 day; and the brain survives for 10^4^ days (100 years). Then, a full dynamic connection matrix for human brain will have at least 10^15^ × 10^4^ entries. Each entry may require some data characterization, so that the nature (location, structural and functional parameters, etc.) of the connection at that time is included. Lets say this data is (conservatively) 10^3^ bytes. Then the size of one full graphical connectome is about 10^22^ bytes. Of course, one instance of a connectome will not provide population statistics across brains—perhaps as few as a 100 instances will do? So the complete connectomic challenge for the brain is of the ridiculous order 10^25^ bytes!

Connectomicists justify their program by claiming that circuits cannot possibly be reconstructed by considering simply the overlap of axons and dendrites (Denk et al., 2012; Kasthuri et al., 2015), but conveniently forget that Ramón y Cajal made a pretty good living by doing just that. Others like Francis Crick have argued that “God is a hacker” and so the search for overarching principles in brain structure and function is an exercise in futility. But again we do not have to look far to find large scale regularities in the design of single neurons or the circuits they form. These regularities are prominent in the stereotyped patterns of interneuronal connection that are conserved across brain systems areas and particularly so in the neocortex and its connections (Douglas and Martin, 2004, 2007). Of course, this is not to claim that the neocortical circuits in these different species and in different cortical areas are identical, but we can already compile a long list of rules of connectivity that contain the elaborations of the basic themes seen across areas and species. The important consequence of such regularities is that they offer the means for a huge compression of connectomics data. Therefore, determining where such regularities exist, and describing their forms and frequencies, should be the first strategic action of any large-scale neuroanatomical research program. It is thus astonishing (to us) that there are few qualitative descriptions of cortical circuits (albeit even Ramón y Cajal was challenged to do this) and vanishingly few quantitative data describing cortical networks in different species and areas. The studies such as Binzegger et al. (2004) and Oberlaender et al. (2011) are amongst the very few attempts to map comprehensively the average connections at synaptic resolution of any cortical area. For a zettabyte of reasons, nothing that has yet emerged from the “dense” or “saturated” reconstructions of tiny patches of neocortex of one mouse has provided anything close to such a comprehensive picture of the average connectivity of a cortical area.

While we wait in anxious anticipation of the breakthroughs needed to automate the process of obtaining even partial reconstructions at synaptic resolution, we note the paradox that the zettabytes of detailed structural and functional data that are considered necessary to understand the structure and operation of the brain stand in stark contrast with the approximately 1 gigabyte of genetic information available to the embryonic cell in order to construct an entire animal, including its brain. Fundamental work on complexity and data compression (Chaitin, 1977) tells us that it is not possible to create an object that is more complex than the total information of the source program and data that defines its construction. Thus, we can expect that the actual complexity of the brain is many orders of magnitude less than the exhaustive connectomics programs imply, and thus the proposed exascale connectomes are likely to be hugely redundant. The apparent complexity of neural connectivity may be more easily grasped than currently believed or implied by the sheer size of the data storage calculated for connectomic projects.

Our line of reasoning suggests that a much more profitable approach is not brute force dense reconstruction of partial circuits in individual animals, but instead to identify the principles of connectivity from the point of view of a self-constructing connectome Zubler et al. (2013). The development of neocortex is particularly interesting in this regard, because the regularities of structure discussed above raise questions of how apparently complex neuronal connectivity could be established on a basis of relatively simple developmental rules enclosed in only a few precursor cells. Moreover, because the cortex is critically involved in intelligent behavior, an understanding of its self-construction is expected to yield insights into the principles of processing in those circuits useful both for neuroscience, and for developing neurally based computational technologies.

### General comments and discussion

#### DeFelipe

I fully agree with Douglas and Martin that the data obtained from the so-called dense or saturated reconstructions of small pieces of the neocortex from a single mouse is difficult—indeed impossible—to interpret in terms of determining the general scheme of connectivity of a given cortical area (see also Markram et al., 2015). The strategy should be to find general rules of connectivity based on sparse data obtained from several samples and animals. In my opinion, we now have to adopt two approaches to better understand brain organization: (1) determine the basic bricks of brain organization which are common to all mammalian species, and (2) determine the fundamental structural and behavioral aspects that are unique to humans, which obviously should be dealt with by studying the human brain directly. The first approach is essential at present since—for obvious ethical reasons—many of the necessary datasets on brain organization cannot be obtained directly from human brains. Thus, discovering the common characteristics of brain organization and measuring these features in a detailed quantitative manner are critical goals. For this purpose, it is not necessary to get the data from all cortical layers and areas of a given species to find out the general rules. On the contrary, if a given property is found in different functional regions such as motor, somatosensory, visual, frontal, parietal, or temporal areas of a variety of species like mouse, rat, cat, and monkey, then it should be safe to assume that this same property is a basic principle of the cerebral cortex in general and that, in principle, it could be applied to all mammalian species, including humans. For example, pyramidal cells do not constitute a homogeneous group of cells from the anatomical, molecular and physiological points of view, as there are clear variations in these properties depending on the cortical layer, area, and species studied (see DeFelipe's comments on the article by Ed Lein below). However, there are geometrical principles, connectivity rules and neurochemical and physiological characteristics that are common to all of them or to subpopulations of certain pyramidal neurons located in the above-mentioned cortical areas and layers in all the species listed. Thus, if we could obtain detailed quantitative data about these general features and understand the functional significance of these regularities in a given experimental species, these findings could be used as predictive neuroscience to be extrapolated to all mammalian species. In this way, it would be possible to create a general model of cortical circuit with no need to obtain the data at all levels of analysis and resolution obtained in the same experimental animal. However, we have to be careful to avoid oversimplifying. For example, it has been shown recently that human pyramidal neurons are not “scaled-up” versions of rodent or macaque neurons, but have unique structural and functional features (Mohan et al., 2015). Similarly, species differences have also been found regarding basket cells and chandelier cells, which are the major sources of inhibitory perisomatic synapses on pyramidal cells. For example, these interneurons can be defined as fast-spiking, parvalbumin-positive GABAergic cells, but Povysheva et al. (2013) found that in the rat and monkey there are differences between basket cells and chandelier cells regarding certain electrophysiological membrane properties and that some of these differences were species-independent, whereas, others were species-specific. Finally, neuronal elements are differentiated into subtypes, some of which are lacking or highly modified in different cortical areas or species and consequently not all cortical regions in all species have the same neuronal elements (e.g., see DeFelipe et al., 2002). Thus, this general model of cortical circuit should be considered as a starting point that should be validated and “adapted” to particular cortical areas and species.

#### Rockland

##### General comments on DeFelipe's comments

Are “dense reconstructions” essential to advance understanding of cortical circuitry? Are they desirable? Both Douglas and Martin in this article, and DeFelipe in his commentary take a critical view and argue in favor of what could be called “directed” or “discrete” (DeFelipe, 2015) sampling. In an early example of this approach, Megias et al. (2001) exhaustively charted the total number of synapses on a sample of hippocampal CA1 pyramidal neurons, utilizing combined light and electron microscopy. There have been surprisingly few similar studies, probably because of the labor-intensive nature of the techniques, coupled with, for many years, the relative lack of interest in detailed morphology by the funding agencies. Given recent technical improvements, this combined approach will hopefully attract renewed interest. What is currently (artificially?) perceived as two, opposing approaches may yet be productively reconciled.

It is quite true, however, that there are “vanishingly few quantitative data describing cortical networks in different species and areas” (Douglas and Martin). In other words, the field needs *both* dense reconstructions and discrete sampling, *and* particularly, more “complete” data. Neurons, as Douglas and Martin state, may be a useful level of quantification; but for most purposes, they are a reduced preparation. Significantly more information results if “neurons” (usually equated with cell bodies) are understood in the context of their axonal arborization (Figures 5–7 in DeFelipe's target article), *plus* their molecular, genetic, and epigenetic specifications and interactions. This returns us to the need for an “integrative approach,” a second “solution” emphasized in DeFelipe's target article.

Finally, a comment on the three p's: properties, principles, and predictions. “Principles” are often stated as the desired outcome, leading to successful predictions; but I wonder if in fact, at this still very elementary stage, there shouldn't be more emphasis on “properties”? After all, it was the understanding of individual properties and their orderly change that lead to the “principles” of the Periodic Table. In addition, there is the fundamental fact that the brain does not exist in isolation (Figure 1 in the target article). All this is undoubtedly immensely challenging, but need be no more discouraging than other “moonshots” that have been attempted, some with conspicuous success.

#### Shepherd

Javier DeFelipe has done a great service in focusing attention on the sheer size and complexity of the anatomical connectomics of the brain. I would like to second the motion of Peter Somogyi that the anatomical problem cannot be studied in isolation from the problems of the functional complexity, molecular complexity, and all the other levels of complexity underlying brain function. I'd like to add: nor should it be made in isolation from the perspective of evolution.

In this regard, a key point is made by Rodney Douglas and Kevin Martin: in reducing and making more manageable what seems like an overwhelming complexity of data, what we should be looking for is “regularities” in the anatomical patterns at all levels—synaptic connections, parts of neurons, whole neurons, whole neuron projections, interneuronal connections, and interregional connections. Our long-term strategy in the search to identify these regularities has been to come at them with an evolutionary approach, starting with the simplest forebrain cortex represented by the olfactory cortex. The “basic circuit” emerging from that analysis turned out to be echoed in the basic circuit for hippocampus, and further in the basic circuit identified in the dorsal cortex of reptiles. This suggested the hypothesis that this consensus cortical circuit could constitute a basic circuit that was amplified in the multiple layers and cell types of the neocortex. The canonical circuit coming independently from their studies of the visual cortex expressed these same regularities of circuit organization.

The advantage of the basic circuit/canonical circuit approach is that, as Douglas and Martin point out, “they offer the means for a huge compression of connectomics data.” They also offer the means for putting the current work on neocortex in an evolutionary context, in which the complexity of the neocortex can be seen to be an elaboration of its evolutionary “bauplan.” My paleontology colleague Timothy Rowe has taken that problem back to the origin of the mammals, around 250 million years ago. Analysis of endocasts of these first mammals evolving from reptilian-like predecessors has provided evidence that the forebrains were dominated by olfactory cortex. The implication is that the regularities that emerged in the earliest neocortex reflected three-layer cortical antecedents in reptiles, as well as olfactory dominance in most mammals from their earliest appearance.

Wider use of this approach could greatly enhance the efforts to reduce the complexity of the neocortex, one of the chief challenges laid down by Javier DeFelipe. It will have the additional advantage of placing current connectomics in an evolutionary context, satisfying Dobzhansky's maxim: “Nothing in biology makes sense except in the light of evolution.”

#### Douglas and martin's response to shepherd's comment

Dobzhansky's is a comforting aphorism that probably no biologist would deny. Evolutionary theory, however, describes only the stochastic search behavior of biological mechanisms. We argue (more fundamentally) that by understanding the principles of self-construction exhibited by the mechanisms of brain development, we will have a better chance of explaining the reliability, regularity, and evolutionary innovation inherent in cortical/brain circuitry.

#### Shepherd's response to douglas and martin's response to shepherd's comment

We agree that nothing in biology makes sense except in the light of self-construction during development. Together, the two aphorisms define the dual role of evo-devo in the functional organization of the brain.

## Identifying design principles to predict brain structure and function

### Sean L. hill

The position taken by Javier DeFelipe comes at an important moment for neuroscience. A debate is coursing throughout the global theoretical, computational, research, and clinical neuroscience communities as large-scale initiatives emerge worldwide promising to tackle the complexity of the brain and its diseases with a diverse array of technology development, data measurement, data integration, modeling, and simulation. The era of Big Data is fundamentally impacting the path of modern neuroscience.

Principles to predict connectivity—there is a clear need for a theory of connectomics (i.e., structural principles that can predict specific functional properties) enabling specific predictions for example of how dendritic spine shape, active zone geometry, postsynaptic density, mitochondrial size, etc.—determine synaptic strength, short term depression and facilitation, long term plasticity and homeostatic processes. DeFelipe provides important concrete examples of elements of such an approach. Of course other principles clearly may come into play including the dynamics of ion channel and receptor localization and dynamics within the membrane of the neuron itself. However, making a concerted effort to map out these core structural principles has the potential to make a tremendous impact on the grand challenge of whole brain connectomics. Even the beginnings of such a theory could alter for example the specific tissue preparation protocols and provide targets for selecting key features in EM data that could speed the processing of these immense datasets.

Currently, we are witnessing the beginning of a tsunami of single cell transcriptomic data which is serving to form the foundation of data-driven taxonomies (Sugino et al., 2006)—and will likely lead to data-driven ontologies with the specific prediction of morphological, electrophysiological, synaptic, and connectomic properties. In addition, such data is already at the core of new algorithms that predict the composition and spatial distribution of cell types throughout the brain (Grange et al., 2014) when combined with whole brain gene expression atlases (Lein et al., 2007).

Multi-modal—and multi-scale—data integration promises to help form an integrative view on the structural and functional organization of the human brain (Amunts et al., 2014). But in addition, cross-modal and cross-scale studies hold the promise of enabling large-scale prediction of cellular and synaptic level connection properties. As DeFelipe points out, when a presynaptic axonal swelling forms an apposition with a postsynaptic process on a dendrite within 0.5 μm under light microscopy—this putative synapse stands an 80–90% chance of being a verifiable functional synapse (i.e., with clearly defined presynaptic vesicles, active zone, and postsynaptic receptor density) in electron microscopy. Even this rough estimation can provide a valuable picture of the potential circuitry—an important basis for characterizing whole cellular and microcircuit connectivity which is not anticipated to be possible for many years using EM imaging alone.

Computational models of microcircuitry (formed by distributing hundreds or thousands of 3D cellular morphological reconstructions to statistically reconstruct the cellular structure of a local brain circuit) can also provide an important tool to gain insight into the principles underlying brain construction. For example, a recent computational study predicts that the role of the great diversity of individual neuron morphologies in a somatosensory cortical microcircuit (i.e., the fact that no two neurons have the exact same branching structure) is to ensure that all neurons in the microcircuit have invariant distributions of input and output synaptic locations independent of cellular density and specific positioning (Hill et al., 2012). Thus, morphological diversity is predicted to be essential to forming a robust cortical wiring diagram although constructed by a biological process that results in a high degree of variability.

Identifying the relationship between the structural locations and properties of synapses and dendritic spines and the postsynaptic response is also an essential link in predicting functional properties from anatomical and structural studies of brain circuitry. A related computational study to the one above found that the shapes of the neurons dendritic and axonal arbors and the resulting potential locations for functional synapses could predict the distribution of postsynaptic potentials observed in *in vitro* studies (Ramaswamy et al., 2012). Even more explicitly, new data-driven tools have been developed to map the relationship between specific morphological properties of whole human neurons and their dendritic spines and specific neuronal and synaptic functional properties (Toharia et al., 2016). Such predictions are important steps to understanding fundamental principles relating structure to function at the synaptic level.

Finally, it is now becoming possible to predict network dynamics by integrating many levels of data (including gene expression, ion channel kinetics, cellular firing properties, neuron morphology, synaptic dynamics, and connectivity properties) into data-driven mathematical models and simulating chemoelectric activity and interactions using a supercomputer (Markram et al., 2015). This recent study illustrates that such computer models can provide an important tool in understanding the structure and function of brain circuitry and the relationship between specific ionic and neuromodulatory factors in determining network states and the response to stimuli.

While these examples support DeFelipe's thesis across multiple scales of organization in the brain, it is of course essential to recognize the dramatic limitations in our current models and their ability to predict all the levels of complexity in the brain. Clearly the brain is a remarkably dynamic entity and the snapshots provided by even the best data generated today are remarkably sparse both spatially and temporally. Examples of the dynamic structure of brain circuitry are plentiful: with axonal sprouting, dendritic arbor changes, and dendritic spines appearing and disappearing within minutes. Additionally, even cellular genetic identity as identified through single cell transcriptomics looks to be vulnerable to activity dependent changes as shown in a recent study (Dehorter et al., 2015). Other studies show that as the brain changes state from wakefulness to sleep the extracellular space and geometry of glia can dramatically change and reconfigure to perform physiological cleaning functions.

Yet, the only way forward is to start to capture even the first order principles to make sense of the complexity and to lay the foundation for exploring the next levels of complexity and identifying the organizing principles that will drive our ultimate understanding of the brain.

### General comments and discussion

#### DeFelipe

The article by Sean Hill not only highlights several interesting topics such as predictive neuroanatomy and multi-modal—and multi-scale—data integration, but also his comment on the dynamic structure of brain circuitry made me think about the controversy that began back in the 1890s about whether or not the structure of neurons remained fixed over the course of an individual's life. The debate centered on whether the complex processes of the neurons and the neuron-to-neuron connections were permanent and unchanging over time or were the neurons and their connections dynamic and constantly changing? As Hill clearly stated, now we know that brain circuitry is dynamic. One of the consequences of these ever-changing circuits is that this leads to an increase in the variability of the data obtained when examining the brain at the structural, neurochemical or physiological levels. However, it is also true that data obtained in several studies at different ages show that this variability remains within a relatively narrow window. Thus, there are biological rules that constrain the dynamic changes of the circuits and these rules can be determined, making it possible to build computational models of brain circuits.

#### Rockland

I strongly agree that the inherently dynamic property of the nervous system needs to be considered a core feature, and that further investigation is likely to be a fruitful direction [for example, does the variability really remain within a “relatively narrow window” (DeFelipe's comment)]. Not only is “brain circuitry” dynamic, but very likely so are many other aspects as well. Thus, an especially important issue is how we extrapolate from the typical “snapshot results” to the actual, “remarkably dynamic entity.”

Sean Hill has nicely summarized the present state of the field; namely, serious (“dramatic”) limitations in our knowledge, but a clearly perceived need to advance from “first order principles” to “exploring next levels of complexity and identifying …organizing principles.” This is an important reminder that “principles” come in different flavors, some more like stepping stones on the way to something more foundational.

## Refer to the blueprint

### Ed S. Lein

Despite remarkable progress in neuroscience over the last decades, we find ourselves incredibly far from a deep understanding of human brain structure and function and how this complicated system processes information to give rise to our wide spectrum of mental faculties. The rise of model organisms for studying brain architecture, in particular the mouse with its range of options for genetic targeting and manipulation, has dramatically accelerated the study of conserved features of mammalian brain organization but leaves largely unaddressed a fundamental problem: how similar is the rodent brain, or for that matter the non-human primate brain, to the human brain and how far can we push these models as proxies for studying the human brain itself? The generally dismal experience of the pharmaceutical industry in the use of the mouse as a preclinical model provides a sobering backdrop for the premise of species conservation, and thus we find ourselves with a dual problem. First, the complexity of the brain and the challenges associated with bridging levels of resolution from macroscopic through microscopic present a seemingly overwhelming challenge for today's technologies, as eloquently laid out by DeFelipe's (2015) thoughtful discussion. Second, in order to understand the human brain there is a critical need to study the human brain itself, with its limited experimental options and tissue availability, or at the least to validate the conservation of features across species.

Despite these challenges there is great cause for optimism on many fronts. Firstly, there is growing appreciation and funding for large-scale, multidisciplinary efforts that combine major data generation projects, informatics efforts for data integration, and computational modeling. Secondly, there is increasing emphasis, with dramatic gains, on generating new experimental and analytical tools to drive progress in what has been a fundamentally technology-limited domain. Thirdly, neuroanatomy as a discipline is resurgent with the tools to define and study selective cellular elements of neuronal circuits and the growing recognition that describing the connectome or wiring diagram of the brain is essential to understanding its function. Fourth, transcriptomics is finally coming into bloom as the means to study the genetic code underlying brain development, structure, and function through its application at the proper level of resolution: specific cell types and individual cells, the units of transcription. These latter anatomical and molecular techniques are increasingly scalable, leading to a realistic outlook for reasonably comprehensive descriptions of cell types and the circuits they make up given sufficient resources. Finally, many of these techniques are applicable to human brain, even including functional analysis in *ex vivo* tissues from neurosurgical resections.

So where would the neuroscience community's efforts best be placed to tackle the problem of brain complexity? In deference to the arguments of DeFelipe (2015) for creative data sampling and modeling, there is also a great need for large-scale, “near comprehensive” data generation and modeling efforts. While giving lip service to the enormous complexity of the brain, most modern neuroscience is nevertheless performed on a small scale and the results quickly oversimplified and overgeneralized. We do not begin to have a good description of the properties of the roughly 86 billion neurons in the human brain (Herculano-Houzel, 2009) or the larger circuits they make up through selective connectivity. The issue of quantitatively defining cell types and their connections is fundamental to the entire problem of brain complexity. How can we hope to understand the function of this system without an understanding of its parts? How can we generalize and integrate findings within and between laboratories if we cannot be certain that we are measuring the same entities? And how can we know how much to simplify our models without first examining the details of the system to understand what is essential?

But how to approach the problem? Starting with cellular anatomy? Physiology? Genes? The former two have been the traditional approaches yet have proved quite limited in their ability to unambiguously and quantitatively discriminate among neuron types while largely failing to provide a broad conceptual framework for cell type classification. On the other hand, the utility of gene expression for understanding brain structure and function in a broad conceptual sense has been rather limited until recently as well, despite large scale efforts to map gene usage across the adult and developing brain (Lein et al., 2007; Hawrylycz et al., 2012). In the realm of cell type classification, gene expression has taken a back seat to morphological and electrophysiological characterization except as markers of broad cell classes. However, recent advances have changed this equation. Measured in toto at the level of relatively homogeneous zones (Bernard et al., 2012), isolated cell populations (Sugino et al., 2006; Doyle et al., 2008), or individual cells (Macosko et al., 2015), the rich tapestry of the complete genetic code provides something different that may prove transformative: a quantitative framework for understanding the complete cellular makeup of the brain. Perhaps not surprisingly, the transcriptome with its 20,000+ elements that code for all cellular functions tends to vary more substantially between cell types than other measurable cellular features, and allows a purely data-driven genetic classification of circuit elements (Macosko et al., 2015; Tasic et al., 2016). Several studies have shown that transcriptome similarity varies by developmental origin as well (Zapala et al., 2005; Bernard et al., 2012), perhaps at least roughly proportional to descent from a common progenitor cell. How discrete transcriptomes of similar cell types will be, and how well other cellular modalities correspond will be essential to demonstrate, yet it is not unreasonable to hypothesize that genes will covary with the properties they underlie in the same cell.

Ironically, then, the best way to tackle the “anatomical problem” of DeFelipe (2015) may be to start elsewhere, with the genetic blueprint, and layer on information of various types at various scales onto this “molecular taxonomy” of cell types (Sugino et al., 2006). Transcriptomics may provide a sorely lacking framework for organizing information about brain circuit organization at the level of its constituent parts. Cellular elements are fundamentally defined by their developmental origin and genetic specification, and ultimately take on their specific mature molecular, anatomical, connectional, and functional properties through some combination of intrinsic and contextual factors to form local and long-range functional circuits. This is of huge practical significance, as single cell transcriptomics is highly scalable and suitable for large-scale efforts in adult and development. Furthermore, this can lead to a common molecular and cellular language: discriminatory molecular signatures for specific cell types that the community can use to link results across laboratories, both in model organisms and human.

Paradoxically, the fields of neuroscience and neuroanatomy are both overwhelmed with bits of data and incredibly data starved for enough of the right types of data. The complexity of the human brain, with its ~86 billion neurons (Herculano-Houzel, 2009) and 100 trillion connections, is not in my opinion a small problem that will be solved by small-scale selective sampling, any more than the genome with its 3 billion bases could be solved by sequencing a small number of individual genes. We need a big data—omics mindset to tackle the complexity of the most complex piece of matter in the known universe, coupled with strong efforts in informatics, and modeling, to create a scaffold for organizing information akin to the physical scaffold of the genome (Lander et al., 2001). Such foundational efforts to better understand the complete blueprint of the normal brain are essential to allow an integration of data at many scales, and provide a conceptual, data and informatics framework moving from genes to cell types to physical circuitry to functional circuitry in the context of behavior. The genetic blueprint is a great place to start.

### General comments and discussion

#### DeFelipe

In my opinion, the optimistic view expressed by Ed Lein regarding the goal of understanding the human brain is well-justified and expounded and brilliantly emphasizes the importance of discovering the transcriptome organization of the brain. In the field of single cell transcriptome work, the term “cell types” is commonly used to refer to selective subpopulations of neurons, rather than specific cell types. For example, “layer V pyramidal neurons” are physiologically and anatomically heterogeneous and include different subtypes that are generally grouped into two main types: thick-tufted (or type I) neurons that project to subcortical targets such as the striatum, superior colliculus, brainstem, and spinal cord, and that discharge a short burst of spikes; and thin tufted (or type II) pyramidal cells that project to the contralateral cortex and the ipsilateral striatum, and that discharge spikes with no adaptation. In addition, different classes of tufted populations have been identified on the basis of morphological and electrophysiological characteristics, or on the basis of the expression of different combinations of neuronal markers (e.g., transcription factors and neurofilaments) (e.g., Molnar and Cheung, 2006; Porrero et al., 2010; Antón-Fernández et al., 2015). Thus, layer V pyramidal neurons can be further morphologically, neurochemically, and physiologically characterized in such a way that knowing an incomplete set of different combinations of features may serve to predict the remaining molecular, morphological, electrical, or synaptic characteristics of the cells under study.

A good example of this predictive approach would be the observation by Tyler et al. (2015) that layer II and III pyramidal neurons show different electrophysiological and structural properties depending on their precursor cell type of origin. These authors have found that both the morphological complexity and certain basic membrane and action potential firing properties of layers II and III pyramidal neurons are, in part, specified at birth by their progenitor class of origin. More specifically, they observed a lower apical dendritic branching complexity and higher input resistance in Tbr2 vs. non-Tbr2 lineage neurons. Thus, the identification of proteins or transcription factors that are differentially expressed in the Tbr2 and non-Tbr2 lineages could be particularly useful to characterize large collections of neurons labeled with intracellular injections of markers such as Lucifer Yellow (LY) that are available in the cerebral cortex of the adult mouse, rat, and human cerebral cortex. These neurons can be reconstructed in 3D to analyze their morphological attributes. The histological sections containing these cells can be processed for immunohistochemistry to try to correlate the differential molecular expression with the morphometric parameters of the pyramidal cells. The morphometric data obtained can then be extrapolated to other cortical regions where genetic/molecular mapping is available but not for the morphology of pyramidal cells.

#### Rockland

Three things jump to mind. First, the call to optimism in the original article, as reinforced by DeFelipe: I concur. The challenge—of understanding the brain - is inarguably significant and important; and the tools available—from transgenic monkeys to small-brains-in-a-dish—are already impressive and getting better. Even the acknowledgement that we have far to go, in understanding the brain, can be seen as a definite positive. Second, the issue of model systems is now complex. What, in this age of CRISPR, can mice, drosophila, or non-human primates contribute to fundamental understanding of the human brain? Probably all can contribute something, but there is good reason to tread carefully. Third, what about the important issue of cellular classification? Is Ed Lein correct that “the genetic blueprint” will provide a framework and answer to the “anatomical problem”? Possibly, but other factors, such as epigenetic regulation, alternative splicing, and posttranslational modification contribute to neuronal diversity (Erwin et al., 2014). DeFelipe's commentary again repeats the desideratum that “combinations of features may serve to predict the remaining molecular, morphological, electrical, or synaptic characteristics of the cells under study.” But one also has to take account of “location,” in relation to significant heterogeneities in brain architecture; for example, callosal and acallosal regions within primate V2 or other areas.

## What changes do we need?

### Kathleen S. Rockland

Evidence suggests that the field of neuroscience is entering a new stage. “Big data” and the search for comprehensiveness (i.e., the various “-omes”) figure prominently in what has all the signs of a new culture, if perhaps not yet a major paradigm shift. If this is the adolescence of neuroscience, it may not surprise that it comes with a certain amount of confusion and anxiety. Thus, there is at least a temporary downside, succinctly captured by DeFelipe's (2015) thoughtful discussion on “how to deal with the problem of imprecise connectomes and incomplete synaptomes.” As DeFelipe proposes, an obvious approach (“potential solution”) is modeling or simulation, inspired by selective sampling of the available data, in turn, guided by “rules” derived from decades of previous research.

I would add to this a corollary approach; namely, distorting the known facts, and perturbing accepted “rules.” For example, what happens to simulations if the dendritic spinefree zone, proximal to the pyramidal cell soma, is populated with spines? If pyramidal cell somas are (incorrectly) modeled with both excitatory and inhibitory synapses, or with varying numbers of inhibitory synapses? If all the modulatory connections are specified as serotonergic (or noradrenergic or dopaminergic)? If hippocampal CA1 is populated with CA3 neurons (characterized by long associational collaterals and thorny dendritic excrescences), etc.?

Deliberately skewed simulations might also address the problem of variability, at the level of cells as well as brains (i.e., the issue that “there is no bridge between brains; all species have different brains,” DeFelipe, line 275). For example, in the rodent barrel cortex, mice have “hollow” barrels, but rats have “solid.” Could simulation carry out a cross-species “transplant” and detect functional consequences?

The “magnitude of the problem” (DeFelipe, line 090) refers in part to the sheer, overwhelming amount of data. It also alludes to the overwhelming complexity of the brain. Curiously, despite wide agreement that the brain is complex, the neuroscience field as a whole often seems to prefer an assumption of uniform and stereotyped organization, to the extent that a field-wide tendency for premature simplification can be considered another major problem (see G.M. Shepherd's Einstein quote: “Everything should be as simple as possible, but not simpler”). Species and structures *are* different (DeFelipe, 2015), and the differences can be provocative, informative, and illuminating.

At least some research areas, such as the investigation of cellular subtypes, have served to counter the urge toward uniformity. The issue of neuronal subtypes now extends to differences in developmental history and molecular signatures. Related, synaptic diversity and “connectional weights” are being examined in the context of populational coupling, and interpreted as a range of types, from strongly coupled “choristers” to weakly coupled “sololists” (Okun et al., 2015).

Neuroanatomical images are especially effective in spotlighting variability and complexity. The single axon images included by DeFelipe vividly demonstrate that individual axons connecting the same source and target are not “point to point,” but rather have important qualities of divergence and convergence. Moreover, within a defined connectional set, the axons are not stereotyped. The more interesting interpretation is that this is not “random variability” but rather is necessary and deliberate, for reasons yet to be established.

Surprising or puzzling results (i.e., those that don't fit into standard, too often dogmatic views) should not be ignored, but rather highlighted as important clues. Thoughtful formulation of “why” questions can be as helpful, if not more so, than “hypotheses.” For example, why is it that in human (but not rodent) brains different types of glia tile the space in a non-overlapping manner, while others are more overlapping (Oberheim et al., 2006)? Why are there both topographic and non-topographic connections (respectively associated with cortical layer 4 and layers 1, 2; Rockland, 2015)? Why is there modularity in neocortical layer 2 (Ichinohe et al., 2003)?

The temptation toward simplification, insofar as it is a problem, may call for a change in ethos, where the field as a whole is willing to acknowledge and engage details; that is, in the present context, neuroanatomical details. Without that, generalizations such as “the thalamus” or “the cortex” have a dangerous averaging effect, with meaningless blurring of substantial differences of sensory/motor/associational regions, each with distinctive developmental, neurochemical, connectivity, and species features.

The solution? One thinks of a cultural climate in the discipline which will be tolerant of the enormous problems and limitations of current approaches, while not losing sight of future promise. More specifically, much of this may translate into renewed valuation of teaching, especially at the early level middle or high school. A priori, there seems no reason why neuroanatomical structures and pathways could not beneficially be taught, even like the foreign languages, at a relatively early stage. As it is, neuroanatomy—once recognized as the “language” of neuroscience—is only minimally taught outside of the medical school curriculums, and drastically pared down within these.

As a final point, I will mention that the program of connectomes and other—omes, while serving a useful near-term purpose, carries its own problems. “Completeness” is neither realistic nor desirable. It's easy to see the unrealistic. Whole neuron analysis, even if mapped to exquisite, EM-level completeness of the full set of individual dendritic spines and synapses, receptors, axon trajectory and diameter, and postsynaptic partners will still fall short in detail alone, not taking account of lifespan history, the temporal dimension, metabolism, genetics, or other factors. As effectively illustrated in DeFelipe's Figure 1, fuller understanding of the brain requires incorporation of non-neural components; for example, the body and the external environment. “Undesirable” is also clear, in that too tight a tether to data (“bottom-up”) is bound to have a stifling effect.

The solution? DeFelipe has put forth the importance of creative sampling and modeling, with the specific call not to neglect the rich treasure-trove of comparative neuroanatomy. I will only add, the need to foster open-mindedness (a favorite route for serendipity) and fresh perspectives, to work hard and enthusiastically while recognizing that progress, as concerns the brain, is something likely to be ongoing well into the long-term.

### General comments and discussion

#### DeFelipe

This article by Kathleen Rockland and the next by Gordon Shepherd neatly summarize several concepts that we should keep in mind when dealing with the study of brain organization. I would also like to comment on one additional issue—the modeling and simulation of brain circuits.

Rockland was wondering what would happen to simulations if the experimentalist is “distorting the known facts and perturbing accepted rules.” For example, how would the results be affected “if pyramidal cell somas are (incorrectly) modeled with both excitatory and inhibitory synapses?” This is an inspiring question that I would like to consider in a more general sense. What is the significance of the modeling and simulation of a given circuit in a particular brain region if this circuit is not complete or if it is a hybrid (i.e., combining elements or data from circuits of different brain regions)? Markram et al. (2015) have shown that digital reconstruction of a cortical microcircuit based on data and architectural principles obtained from juvenile rat somatosensory cortex reproduces certain *in vivo* functional findings that were obtained from different neocortical regions in adult animals, even belonging to other species. Thus, Markram et al. (2015) proposed that these phenomena may emerge from fundamental attributes of the neocortical microcircuit. This is in line with certain studies focusing on pairs of synaptically connected cells in the cerebral cortex of several species. For example, Bannister and Thomson (2006) performed dual intracellular recordings in rat somatosensory and visual cortex and in cat visual cortex and examined the properties of excitatory connections between layer IV pyramidal cells and whether these differed between rat and cat. These authors did not find significant differences between rat and cat neocortex in spite of the functional and architectural differences between these cortical areas in the two animals. Nevertheless, as discussed above (see DeFelipe's comments on the article by Rodney Douglas and Kevan Martin), there are structural and functional attributes that are species-independent, whereas, others are species-specific and these differences or similarities may also depend on the cortical region. Thus, depending on the parameters analyzed, the results could be considered applicable in general or to a given region and/or species-specific.

An additional point to be considered is that the functional significance of many aspects of cortical organization is unknown, and it may be that certain structures simply do not have a function. As pointed out by Rockland (2010), for example, the functional significance of the reticular or honeycomb appearance of thalamanocortical and corticocortical terminations in certain cortical areas and layers and species is currently not known. Cortical architecture can even be notably disrupted and yet apparently remain functionally intact. In this regard, Shepherd emphasizes the importance of examining the functional consequences of the natural 3D organization of microcircuits using realistic models. Using this approach to analyze an olfactory bulb microcircuit, Migliore et al. (2014) shed new light on the relations between the functional properties of individual cells and the networks to which they belong. In conclusion, modeling and visualization are useful tools to (1) learn about the data by exploring different hypotheses based on previous knowledge of parameter variations and (2) generate new hypotheses about the structural and functional organization of the brain.

#### Rockland

Three brief comments following on DeFelipe's:

First, about the old problem of structural-functional correlations. As DeFelipe implies, this is a deceptively difficult issue, since “function” is at best only partly understood and since, barring basic reflexes, there are likely to be multiple structural substrates. Ocular dominance columns, their presence or absence, and variations, are a classic example (Horton and Adams, 2005); but plausibly, the same conundrums pertain at microcircuitry and other levels of organization. Particularly apt in this regard is Shepherd's call for a revision of the Neuron Doctrine, to take account that “the neuron is itself a complex cellular system, interacting with other neurons [i.e., other complex systems] to form complex meso- and macro-multicellular stystems.”
Second, while DeFelipe here highlights the search for species-independent “commonalities,” in-depth mining of species-specific adaptations can also be an excellent strategy, as in the cross-species study of “hand,” with all its structural and functional specializations. This is all the more so if, as is increasingly possible, we can include data from comparative genetics and phylogenomics. Broad taxonomic inquiry, for example, poses the fundamental question of whether neurons, like eyes, may have evolved independently at least twice (Strausfeld and Hirth, 2016).
Third, given this view of the task at hand (i.e., cross-disciplinary and cross-scale investigations toward new perspectives and hypotheses, as per DeFelipe's closing comments), there needs to be a supportive culture and intellectual infrastructure. This includes large curated databases, as Gordon details, and, as I wrote, continued review and enrichment of the educational programs.

## “Dense digital reconstruction” for challenging “brain complexity”

### Idan Segev

It seems that many of the participants in this communication agree, and many in the field also do, that eventually we will need to have a “dense connectome” or “synaptome” (DeFelipe, 2010; Seung, 2012; Helmstaedter et al., 2013; Morgan and Lichtman, 2013; Mikula and Denk, 2015) for whole brain regions (e.g., the neocortex), and perhaps eventually for the whole brain? Such “micro-connectomics” will serve as an essential step for understanding signal flow and computations performed by particular brain regions and for correct interpretation of experimental data. The question is whether there are important shortcuts for obtaining such a “synaptome”?

Albeit my strong tendency to a priori simplifying the model systems of interest (inspired by the “equivalent cylinder” cable theory that successfully explains the key aspects of dendritic integration, Rall, 1967), in recent years I have become convinced, and I will explain below why, that we will need to go through a painstaking stage of both having the biological “dense connectome” of a whole system and that this will serve as a key reference for the (already existing efforts, see below) of generating and simulating “dense digital active connectomes” counterparts. Without this latter stage, we will never be able to say with confidence that we fully understood the neuro-phenomenon of interest, from the mechanistic basis of devastating diseases to understanding computational functions implemented by a particular brain region.

By saying that we need “an active dense connectome” I mean to stress that the “synaptome,” on its own, will not suffice without adding, on top of it, detailed physiological information (such as synaptic strength/dynamics and specific membrane excitability for the various cell-types composing the system). We will indeed need a “dynome” (Kopell et al., 2014). The latter should include developmental/plastic principles that enable the adaptation of the “generic” structural and dynamic backbone of the system to environmental demands. Huge efforts are being presently made in obtaining the “synaptome” and the “dynome” by several “mega-projects” worldwide (EU, USA, Japan, China), and therefore I am optimistic that in 10 years or so we will be able to record from, and manipulate, hundreds of thousands or perhaps millions of neurons and synapses, and manipulate them during specific behavior, and that we will also be able to fully reconstruct the micro-connectome of large systems (using thousands of parallel scanning beams and automated electron-micrograph reconstruction aided by sophisticated computer-vision algorithms). Together we will have the “dynome” of a whole system at the synaptic level (e.g., of the whole fly brain, the mouse neocortex or even the whole mouse brain).

Several shortcuts have been proposed toward this goal in the present discussion (e.g., by Rodney Douglas and Kevan Martin and by Sean Hill). Here I will elaborate on one particular promising route, “the dense digital reconstruction and simulation” scheme. This scheme enables one to integrate and share anatomical and physiological data under one framework, enabling to include cell types, connectivity pattern and physiological results and to refine it in view of new experimental data. This provides a systematic tool to study the fundamental structural building blocks of the circuit and to numerically simulate circuit activity under various input conditions, and to compare it to its biological counterpart (https://bbp.epfl.ch/nmc-portal/welcome). I believe that such interplay between the detailed digital dynamic simulations of the circuit and its biological counterpart will provide deep understanding on the space of possible states of the circuit and on the key structural and physiological parameters that govern its activity. In that sense, the digital reconstruction is not only a temporary replacement for the comprehensive data, emerging from the “real” micro-connectome and from multi-cell/synapse recordings, but a complementary and necessary step for modeling and understanding this biological data, once it becomes available (see review on the pros and cons of this “biological imitation game” process by Koch and Buice, 2015).

To the best of my knowledge, two teams are presently intensely involved in an endeavor for constructing and simulating dense digital connectomes at the synaptic level—Egger et al. (2014) working the barrel system of the rat and Markram et al. (2015) on its somatosensory cortex. Both groups are using sparse data about the cell-types composing the circuit, the network connectivity and synaptic/spiking activity in a cortical for replicating digitally a dense circuit in a volume of about 1/3 of a cubic mm (with some 30,000 cell and several tens of million of intrinsic synapses). Reconstructing digitally even larger cortical regions is presently being pursued by these two groups. The details on how the two teams go about this mission, each with their different experimental data and different algorithms for generating the predicted “synaptome” and “dynome” can be found in the above papers (see also Lang et al., 2011; Reimann et al., 2015). In both cases, the assumption is that the available sparse experimental data (anatomical and physiological) provides sufficient constraints such that the digital replica is a good-enough statistical approximation for the real biological counterpart. Both teams are constantly refining their circuit model as more data becomes available, and both have made significant efforts to validate their digital replica against experimental data that was not used for building the circuit. Indeed, using their dense simulations, both teams have proposed new explanation for existing data and have provided new predictions for further experiments (see below).

Hereby is a summary of four key benefits which the “dense digital replica and simulations” provide for facing the problem of brain complexity and, eventually, for progressing our “understanding of the brain.”

#### Suggesting minimal constraints required for faithfully capturing the biological circuit

As pointed out by Douglas and Martin in this discussion, one should expect a relatively small set of principles that underlie the apparent huge circuit complexity. What are these key principles, and how small the set of constraints required to capture the essence of the circuit is yet to be resolved. Indeed, the two dense digital circuits available today are reconstructed using a small set of biological constraints (e.g., the total number of cells in the modeled volume, density of axonal boutons, distribution of cell types/layer, strength/probability of synaptic connection among a selected pairs of neurons, overlap between axons and dendrites as a precondition for generating a putative synapse, etc.). The digital dense reconstruction approach allows for a direct assessment of the impact of each of the constraints used to build the circuit on the circuit's structure and emerging dynamics. But do these constraints faithfully replicate the statistical connectivity/activity of the real circuit? To answer this we will need to have the real dense connectome for the corresponding circuits, preferably few of them per circuit (e.g., for several whisker barrels), together with sufficient recordings under various stimulation protocols from that same circuits. The biological dense connectome will serve as an essential reference for assessing the quality of the digital replica and its underlying assumptions.

#### Developing “network science” inspired by the dense connectome

Even if at present, the two available “dense digital connectome” mentioned above with their accompanied “big data” are just first drafts of the real biological counterpart, they will (and already have) inspire the development of refined “network science” for compactly describing the circuit intrinsic topology. Network science has already provided key insights into other complex networks, including social networks and inter-regional brain connectivity, using notions like “motifs,” “hubs,” “small world,” “rich club” etc. (Sporns, 2010). These studies have demonstrated that such intrinsic network structures might have important functional implications (e.g., for the robustness of the system to various perturbations). However, improved network science is required to capture the intrinsic specificities inherent to neuronal circuits, such as their two major functionally opposing major node (neuron) types—inhibitory and and excitatory—and the many different node subtypes (e.g., the more that 50 morphological cell types and around 10 electrical cell types in neocortical circuit, as in Markram et al., 2015). Such improved network science theory is underway inspired by the existing digital connectomes (Gal et al., unpublished results). We will thus be ready, in due time, to compactly describe using refined network science biologically dense anatomically and physiologically reconstructed neuronal circuits when they become available.

#### Examining and inspiring high-level theories about network dynamics/function

Most present-day high-level theories for the function of brain circuits are inspired by the success of physics to describe complex phenomenon using a set of simple rules. Examples of this approach in neuroscience are “the balance state” theory for explaining the asynchronous activity state of cortical circuits (van Vreeswijk and Sompolinsky, 1996) and the “Reichardt detector” principle (Reichardt, 1961) for explaining directional selectivity in the visual system. Such high-level theories are fundamental for guiding experiments and for extracting simple rules (hoping that they indeed exist) for the network operation as well as for understanding the emergence (across levels) phenomena in the brain.

However, these theories are necessarily based on simplifying assumption (e.g., that neurons could be described as “point neurons,” that their activity could be captures by “integrate and fire” dynamics, etc.). It could well be that these assumptions are indeed sufficient for capturing the underlying mechanisms of the biological phenomenon but, similarly, these theories might completely miss the biological foundation of the studied phenomenon. The digital dense circuit is already serving as an important reference for examining, and even inspiring, such high-level mathematical theories. In particular, examining whether the biological realism already embedded in the dense digital circuit agrees with the existing abstract theories or whether the circuit, with its particular details, partially or completely refutes these theories. If so, then the digital reconstruction might inspire new abstract ideas for explaining the phenomenon of interest.

I would like to reemphasize that we must continue to develop hypothesis-driven abstract theories, as they are absolutely essential for understanding emergent phenomenon in any complex physical system such as the brain. I argue, however, that many existing abstract theories about the brain, although mathematically very elegant (which is a merit on its own), are often too remote from the “call of Biology” and that they will benefit enormously by carefully “listening” to the “dense digital reconstructed connectomes” efforts described above.

#### Suggesting new experiments and explaining existing experimental results

The neuronal circuits studied *in vivo* or *in vitro* provide very limited (and perhaps very biased) access to the details of the circuit of interest. Based on this limited data, the experimentalist may find an interesting phenomenon, e.g., that some cells in cortical circuits act in synchrony with other cells—the “choristers”—and that some are “soloists” (Okun et al., 2015). Such behavior was also found in the dense digital circuit constructed by Markram et al. (2015). This enables one to use the digital circuit for suggesting who among the various cell types are these soloists and choristers and, in particular, what are the synaptic, connectivity and excitability mechanisms that makes these cells behave the way they do. Other examples for a very successful use of the dense digital reconstruction/simulations is the refined understanding of the origin of (the much-used) local field potential (LFP, Reimann et al., 2013) and of the impact of distal inhibitory synapses on the robustness of sensory evoked excitation in the mouse barrel cortex (Egger et al., 2015).

Digital dense active replicas of neuronal circuits are a new tool, a new platform, to be used by both experimentalists and theoreticians in order to tackle the problems posed by brain complexity. It will help to extract key constrains to compactly describe the structural and dynamical features of the “big data” circuit, to interpret experimental results and suggest new crucial experiments, and to inspire novel abstract brain theories that are more closely linked to biology.

## Toward a consensus realistic 3D neuron-based brain

### Gordon M. shepherd

The problem of understanding the complexity of the brain has been expertly presented by Javier DeFelipe (2015). We agree with his diagnosis of the problem. The attempt of much of modern neuroscience research to obtain data to reproduce exactly the complex structures of neurons, their firing patterns, relations to each other, and functions at all levels of organization in hopes of producing insights into “how the brain works,” is proving insufficient, and inexact in many ways. It is becoming apparent that detailing the complexity is the problem, not the solution.

As with every science, theory is needed to give understanding of the experimental data. “You can't understand a fact without a theory” is a mantra from physics. Historically, physics and chemistry have been fueled by vigorous interactions between experimentalists and theorists. Neuroscientists by contrast have traditionally eschewed theory as being soft and vague compared with experimental facts. DeFelipe shows how this has come at a huge cost, of lacking a theoretical basis to explain the facts.

The Neuron Doctrine, the concept that the neuron belongs in the cell theory, established by Ramón y Cajal and his contemporaries in the 1890s, has provided a necessary overall theoretical framework for the neural basis of brain function. DeFelipe joins many of us who believe it needs revision to incorporate new findings, that the neuron is itself a complex cellular system, interacting with other neurons to form complex meso- and macro-multicellular systems.

For half a century we have had clear evidence of the crucial role of theory in modern cellular neuroscience. The model of the action potential by Alan Hodgkin and Andrew Huxley, published in 1952, has played a central role in the rise of modern neuroscience (Hodgkin and Huxley, 1952). Similarly, the compartmental modeling approach of Wilfrid Rall (1964) started the path toward a biophysical basis for integration of synaptic potentials in neuronal dendrites. We brought these two theoretical approaches together in a study of olfactory bulb neurons, in which Hodgkin-Huxley-like action potentials in a compartmental model of a brain neuron led to the prediction of interactions between dendrites, soon confirmed by electronmicroscopy (Rall et al., 1966).

Thus, although neurons are too variable and complex for most analytical mathematical approaches, numerical approaches using computational neuronal modeling provide our best theoretical approach for testing and guiding experimental analysis of neuronal and microcircuit function. However, there is a growing feeling that this approach has not yet led to enough paradigm-shifting breakthroughs. A constant problem is how detailed a neuron model needs to be to be “realistic.” A good guideline is “Everything should be as simple as possible, but not simpler,” attributed to Einstein, but invoked by poets and artists as well. For neuroscience, it means in practice that neuronal and circuit models should be somewhere between including all details (simply reproducing the experimental results) and no details (as in most neural network modeling). The Rall approach follows this guideline, reducing dendritic branching in relation to the problem being studied, and building in only the properties that are essential to elucidate functional principles, as we used in predicting the dendro-dendritic interactions.

A major thrust of DeFelipe's essay is that this “simple but not too simple” approach will be a key for constructing rigorous models that can give insight into principles of organization and function. He rightly cautions against the widespread belief that increasingly detailed reconstructions of the myriads of details of neuronal fine structure and branching morphology is desirable, given the artifacts inherent in the methods. Similarly, he cautions against the widespread belief that building an exact connectome or synaptome of the specific connections between neurons is possible.

Instead, he proposes that the aim should be “to build computational models as simplified abstractions, rather than attempting to fully reconstruct the cerebral cortex or any other brain region.” In practice, this can involve “…(virtual neurons generated by modeling the quantitative morphometric measures of a given population of 3D reconstructed pyramidal cells) with realistic synaptic weights for computational models based on the morphological parameters found in real pyramidal cells …[according to] general anatomical rules …”

This is very similar to the strategy we are using in our current studies of information processing in the olfactory bulb by large mitral cell populations (Migliore et al., 2014). Modeling 500 mitral cells presents precisely the problem DeFelipe discusses: how do we base these models on “simplified abstractions”? No one has labeled the full dendritic branching patterns of 500 cells in a region of interest. Our solution was to take the half dozen cells that have been experimentally labeled and simulate their dendrites by algorithms based on dendritic length, orientation and branching pattern. This generates any arbitrary population of cells with a range of dendritic branching patterns indistinguishable from the “real” neurons. A further problem is that traditional computer simulations do not represent overlapping dendritic branching in true volumes. We therefore construct our cells in 3D, so that branching processes of thousands of cells can overlap and realistically interact within their microcircuits. We provide input to this array from experimental data recording some 200 olfactory glomeruli responding to several dozen different types of odors, producing 4D spatial and activity simulations.

We need to coordinate similar efforts across all brain regions to work toward consensus 4D brains. This will need to be carried out on brains of different species, genders, and at different developmental stages. A key to progress in this integration of experiment and theory is having openly accessible well-curated archives for the models. ModelDB has been designed for this purpose. If models are deposited in large databases such as ModelDB (senselab.med.yale.edu/modeldb), which is now over 1000 curated models, they can be used and adapted by everyone to incorporate new data, and enable consensus on circuit elements, connections, and properties. This supports DeFelipe's recommendation for efforts at the international level, but is also making it possible for all investigators, whether in high profile consortia or working individually, to contribute to the growing consensus.

### General comments and discussion

#### DeFelipe

See DeFelipe's comments on the article by Kathy Rockland.

## A gradual path for discovery

### Gabor tamás

The take home message of the paper is “to link detailed anatomical structural data with the incomplete light and electron microscopy wiring diagrams to build computational models as simplified abstractions, rather than attempting to fully reconstruct the cerebral cortex or any other brain region.” Conceptually this suggestion is indeed timely, however, I would add a little bit on how this might be done. A recent paper in Cell presented the most detailed reconstruction of a volume in the cerebral cortex (Kasthuri et al., 2015) and earlier efforts attempted to map all inputs (chemical synapses) arriving to segments of dendritic trees of identified neurons (Gulyás et al., 1999). In line with DeFelipe's line of thinking starting with Cajal's neuron doctrine, one could develop combined ultrastructural and light microscopic models centered around individual examples of relatively well-known cell types within a reasonable timeframe. Full dendritic trees of pyramidal cells might represent a step too far at the moment, but relatively compact dendritic arbors of some interneuron types could be suitable for a complete map of all chemical and electrical synapses arriving to the cell. This, combined with techniques addressing the neurochemical identity of potentially all inputs—light microscopic superresolution microscopy methods (Dudok et al., 2015) and array tomography (Busse and Smith, 2013) or molecular details of individual neurons such as single cell PCR (Monyer and Lambolez, 1995) and sequencing (Zhang et al., 2014)—might provide building blocks for large scale models of the circuit with sufficient information on variability within and between cell types when iterated.

### General comments and discussion

#### DeFelipe

In my opinion, saturated reconstructions of small regions of the brain as recently published by Kasthuri et al. (2015) is of course a very valuable approach and represents an amazing journey inside the brain networks. However, this approach is very time consuming; according to their own calculations, it takes experienced people about 15 min to trace the approximately 200 cell profiles present in 1 μm^3^ of cortical neuropil. Thus, to reconstruct “only” 64,000 μm^3^, Kasthuri et al. estimated that two experienced people-years of 24/7 tracing would be necessary to segment out all the profiles in this volume. In addition, as discussed above (see DeFelipe's comments on the article by Rodney Douglas and Kevan Martin), the data obtained is unsuitable for statistical analysis as it is based on the analysis of a single individual. Therefore, I think that at present it is impractical to use the strategy of saturated reconstructions to generate meaningful data for modeling brain circuits.

#### Rockland

Here, we return to the ongoing dichotomy between “saturated” vs. “sampled” reconstructions. Actually, Gabor's position, that we should do both, has a lot to recommend it. It should be totally routine to carry out saturated sampling in coordination with more macro-scale analysis. Unfortunately, we return to the issue of partisan camps, artificially separated into dense sampling at a micro level vs. more macro connectivity. One outstanding early example of the combined approach, already mentioned, is (Megias et al., 2001). Much more is needed in this direction.

## Author contributions

JD, RD, SH, EL, KM, KR, IS, GS, and GT contributed to the planning, writing, and editing of the paper.

### Conflict of interest statement

The authors declare that the research was conducted in the absence of any commercial or financial relationships that could be construed as a potential conflict of interest.
